# Know Your Enemy: Successful Bioinformatic Approaches to Predict Functional RNA Structures in Viral RNAs

**DOI:** 10.3389/fmicb.2017.02582

**Published:** 2018-01-04

**Authors:** Chun Shen Lim, Chris M. Brown

**Affiliations:** Department of Biochemistry, School of Biomedical Sciences, University of Otago, Dunedin, New Zealand

**Keywords:** bioinformatics, *cis*-regulatory elements, comparative genomics, non-coding RNAs, pseudoknots, RNA structure prediction, RNA viruses, structural motifs

## Abstract

Structured RNA elements may control virus replication, transcription and translation, and their distinct features are being exploited by novel antiviral strategies. Viral RNA elements continue to be discovered using combinations of experimental and computational analyses. However, the wealth of sequence data, notably from deep viral RNA sequencing, viromes, and metagenomes, necessitates computational approaches being used as an essential discovery tool. In this review, we describe practical approaches being used to discover functional RNA elements in viral genomes. In addition to success stories in new and emerging viruses, these approaches have revealed some surprising new features of well-studied viruses e.g., human immunodeficiency virus, hepatitis C virus, influenza, and dengue viruses. Some notable discoveries were facilitated by new comparative analyses of diverse viral genome alignments. Importantly, comparative approaches for finding RNA elements embedded in coding and non-coding regions differ. With the exponential growth of computer power we have progressed from stem-loop prediction on single sequences to cutting edge 3D prediction, and from command line to user friendly web interfaces. Despite these advances, many powerful, user friendly prediction tools and resources are underutilized by the virology community.

## Introduction

This review illustrates the key concepts and strategies used for prediction of RNA structural elements in RNA viral sequences. A range of RNA structure prediction software and relevant resources are available, but most are underutilized by virologists. Here the concepts and strength of these methods are introduced using examples of successful approaches in viruses, with the intention of bridging the gap. The roles of RNA elements in viral biology is illustrated using well-studied viruses, flaviviruses, influenza, and barley yellow dwarf virus (BYDV). We further review the structures and functions of well-characterized types of RNA elements with the emphasis on prediction approaches and their limitations.

There have been several excellent recent reviews on generally predicting RNA structures, in particular relating to integrating experimental data and on 3D predictions (Cantara et al., 2014; Achar and Sætrom, 2015; Weeks, 2015; Dawson and Bujnicki, 2016; Lorenz et al., 2016; Turner and Mathews, 2016). RNA 3D structure prediction methodology and incorporation of experimental constraint is beyond the scope of this review, but we include examples where they have been utilized.

### Concepts of RNA structure prediction

**Figure 1:**
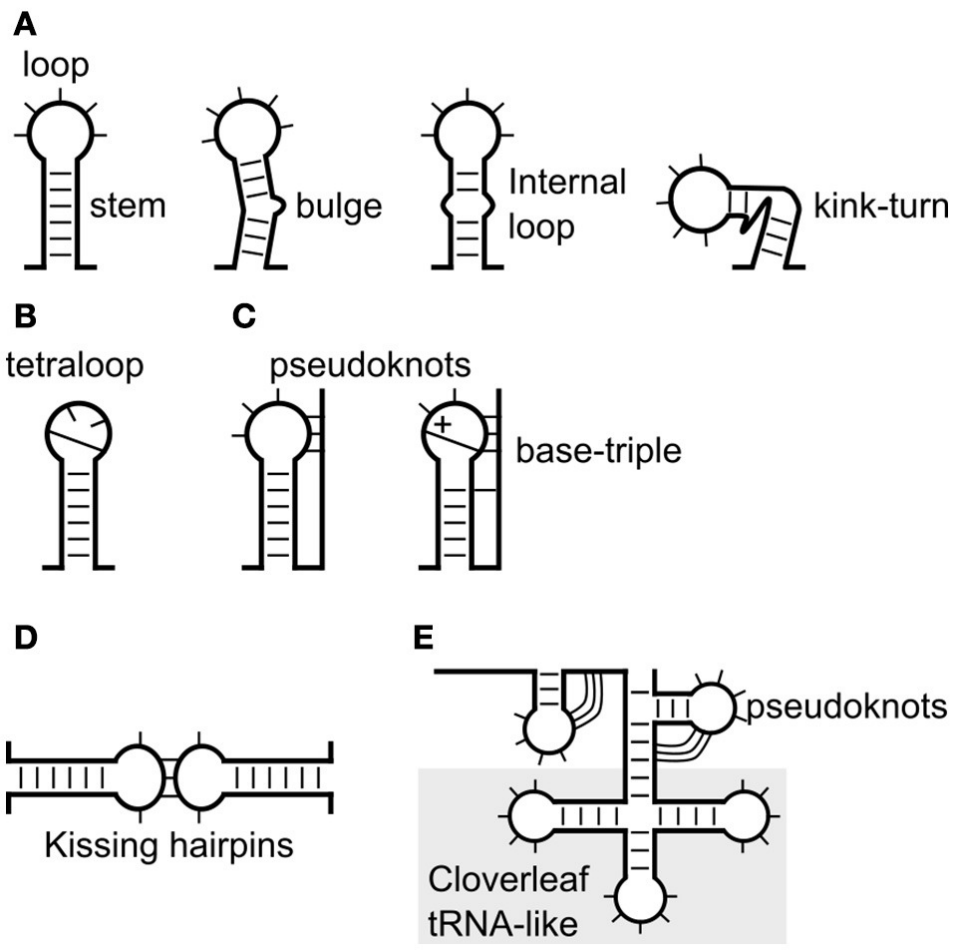
Known viral RNA structures, from stem-loops to complex tRNA-like structures. **(A)** The simplest form of RNA structure is a stem-loop. A stem-loop is shown with a bulge, internal loop or **(B)** tetraloop. **(C)** The loop can also base-pair with upstream or downstream sequences to form a pseudoknot. **(D)** Interaction between the loops of two stem-loops forms kissing hairpins. **(E)** A relatively complex structure is a cloverleaf or tRNA-like structure that often consists of multiple stem-loops and pseudoknots.

Stems involving G-C, A-U, and G-U canonical Watson-Crick base-pairs are the basis of most viral RNA structures, indeed the stem-loop is the basic building block (Table 1, Figure 1). These stems usually form an A-form helix structure, as the 2′-hydroxyl prevents the B-form helix found in DNA. RNA sections with unpaired bases may form structures such as loops or bulges (Bindewald et al., 2008; Table 1, Figure 1). An RNA secondary structure is more likely to be functional if it (i) has a low minimum free energy (MFE) that enables it to fold and base-pair, and/or (ii) is conserved during evolution with covarying stem base-pairs (compensatory base-pair changes). This RNA structural conservation is based on the concept that RNA stems can be conserved regardless of the base-pairs used (Akiyama et al., 2016; Rivas et al., 2017). Both of these features can form the basis of predicting new RNA elements (Xu and Mathews, 2016; Taylor and Hamilton, 2017) and can be integrated with experimental data. Notably, in addition to the fold with the lowest free energy, MFE suboptimal predictions are particularly useful in assessing possible alternative structures of RNA (e.g., pseudoknots; Theis et al., 2008) and long-distance base-pairs (Fricke and Marz, 2016; long-range interactions; Table 1).

**Table 1:** Structural RNA elements, the most used prediction tools, and challenges for their prediction.

**RNA Structures**	**Most used prediction tools**	**Challenges**
**Stem-loop/hairpin**. The helical stem consists of base-pairs. The loop consists of unpaired or non-canonical base-pairs (Zhang et al., 2011).	mfold/UNAFold (Zuker, 2003), RNAfold (Gruber et al., 2008; Lorenz et al., 2011), RNAStructure/Fold (Bellaousov et al., 2013).	Predictions normally only consider standard or canonical base-pairs C-G, U-A, and U-G. Single base-pairs (“lone pairs”) are often excluded by default. Functionally important alternative structures depending on ligand binding need special consideration (e.g., riboswitches).
**Bulge**. A region of a helix where there are no canonical base-pairs at one strand (Zhang et al., 2011).	mfold/UNAFold, RNAfold, RNAStructure/Fold.	Predicted bulges may use non-canonical base-pairs e.g., U-U, A-G, kink-turn.
**Internal loop**. A region of a helix where there are no canonical base-pairs at both strands (Zhang et al., 2011).	mfold/UNAFold, RNAfold, RNAStructure/Fold.	Predicted internal loops may use non-canonical base-pairs e.g., U-U, A-G.
**Tetraloop**. A four-base terminal loop stabilized by intra-loop hydrogen bonds. This stabilizes the stem-loop structure. The GNRA loop is most common, where N represents any base and R represents either A or G (Zhang et al., 2011).	mfold/UNAfold, RNAfold (free energy bonus). RNAComposer (Popenda et al., 2012), 3dRNA (Zhao et al., 2012) (RNA 3D motif prediction).	Most 2D predictions do not predict the intraloop pair (e.g., the G-A pair of the GNRA loop). 3D predictions may predict them. Should be considered if a terminal four-base loop is predicted. Other types of loops e.g., tri-loop and anticodon like loops, can also be stabilized.
**Pseudoknot**. Bipartate structure in which the loop of one stem-loop base-pairs with a sequence outside of the stem-loop (Zhang et al., 2011).	PknotsRG (Janssen and Giegerich, 2015), DotKnot (Sperschneider and Datta, 2010), Pknots (Rivas and Eddy, 1999).	Not predicted by most 2D software. Alternative forms of pseudoknot are found.
**Kink-turn/k-turn**. A three nucleotide bulge in a helix followed by G-A and A-G pairs. Bends the helix (Petrov et al., 2013).	RNAComposer, 3dRNA.	Widespread but most software will not predict these due to non-canonical base-pairs. Requires 3D or homology based software which are yet to be integrated into the most used RNA structure prediction tools.
**Junction**. The point of connection between a number of different helices (Lilley et al., 1995; Bindewald et al., 2008; Zhang et al., 2011).	mfold/UNAFold, RNAfold, RNAStructure/Fold.	Junctions may be important for ligand binding but 3D structures are difficult to predict.
**Base-triple**. A group of three bases which interact by hydrogen-bonds that include edge-edge bonds.	RNAComposer, 3dRNA.	Common in 3D structures.
**Kissing hairpins/kissing loops/kissing stem-loops**. Base-pair interactions between the loops of two stem-loops (Zhang et al., 2011).	pAliKiss (Janssen and Giegerich, 2015).	Difficult to predict without prior knowledge.
**tRNA-like or cloverleaf structures**. Structures with a tRNA-like tertiary structure. In viruses pseudoknots are often located nearby.	Combination of stem-loop and pseudoknot prediction tools.	No specialized tools available to date.
**Long-range intra-molecular interactions**. Base-pair interactions over long distances. Arbitrarily defined as base-pairs over 100 bases apart.	mfold/UNAFold, LRIscan (Fricke and Marz, 2016), CovaRNA (Bindewald and Shapiro, 2013).	Difficult to predict without prior knowledge. Only two specialized tools available to date—CovaRNA and LRIscan. Only LRIscan is optimized for viral genomes and yet to be proven useful.
**Inter-molecular interactions**. Base-pair interactions between two RNA molecules e.g., two copies of a RNA genome.	RNAhybrid (Rehmsmeier et al., 2004), RNAaliduplex (Gruber et al., 2008; Lorenz et al., 2011), bifold (Mathews et al., 1999).	Difficult to predict without prior knowledge.

The limits of current methodology means stems are usually predicted initially using only the canonical base-pairs. However, many non-canonical base-pairs and other structural elements are found in experimentally determined RNA structures (Table 1, Figure 1). About 40% of bases in known crystal and solution structures were either unpaired, or form non-canonical interactions (Stombaugh et al., 2009). Some of the more common non-Watson-Crick pairs in the RNA Basepair Catalog are U-U (about 10% as frequent as A-U pairs, 432 of 4,200) and A-G (about 2% as frequent as C-G, 191 of 9,316; Stombaugh et al., 2009). For example, (i) the base-triple in retroviral encapsidation signals (D'souza et al., 2004) and the base-triples in the pseudoknots of Beet western yellows virus (Su et al., 1999) and Sugarcane yellow leaf virus (Cornish et al., 2005; Figure 1A, and (ii) the kink-turn/k-turn (Figure 1A, Table 1) in the panhandle RNA structure of Influenza A virus that is inducible (Lee et al., 2016) and the A-minor k-turn in the encapsidation signal of Moloney murine leukemia virus (Miyazaki et al., 2010; Table 1, Figure 1).

RNA 3D structures can also be predicted directly from sequences. The accuracy of these prediction tools has improved in the past few years (Miao and Westhof, 2017), such as RNAComposer (Popenda et al., 2012; Antczak et al., 2016), 3dRNA (Zhao et al., 2012; Wang et al., 2017) and SimRNAweb (Magnus et al., 2016). Notably, SimRNAweb has accurately predicted a previously solved frameshifting RNA pseudoknot from beet western yellow virus (Egli et al., 2002).

These predictions can be tested experimentally. For example, to demonstrate that a predicted RNA structure exists and is functionally important, a wild type phenotype can be destroyed with mutations that disrupt the RNA structure (e.g., Fang et al., 2012; Chapman et al., 2014b). This phenotype may be restored by compensatory base-pair changes—changing the primary sequence where base-pairing is still allowed. However, primary sequence motifs and structures of loops and bulges may also have important roles (Bindewald et al., 2008).

### Representations of RNA structures

**Figure 2:**
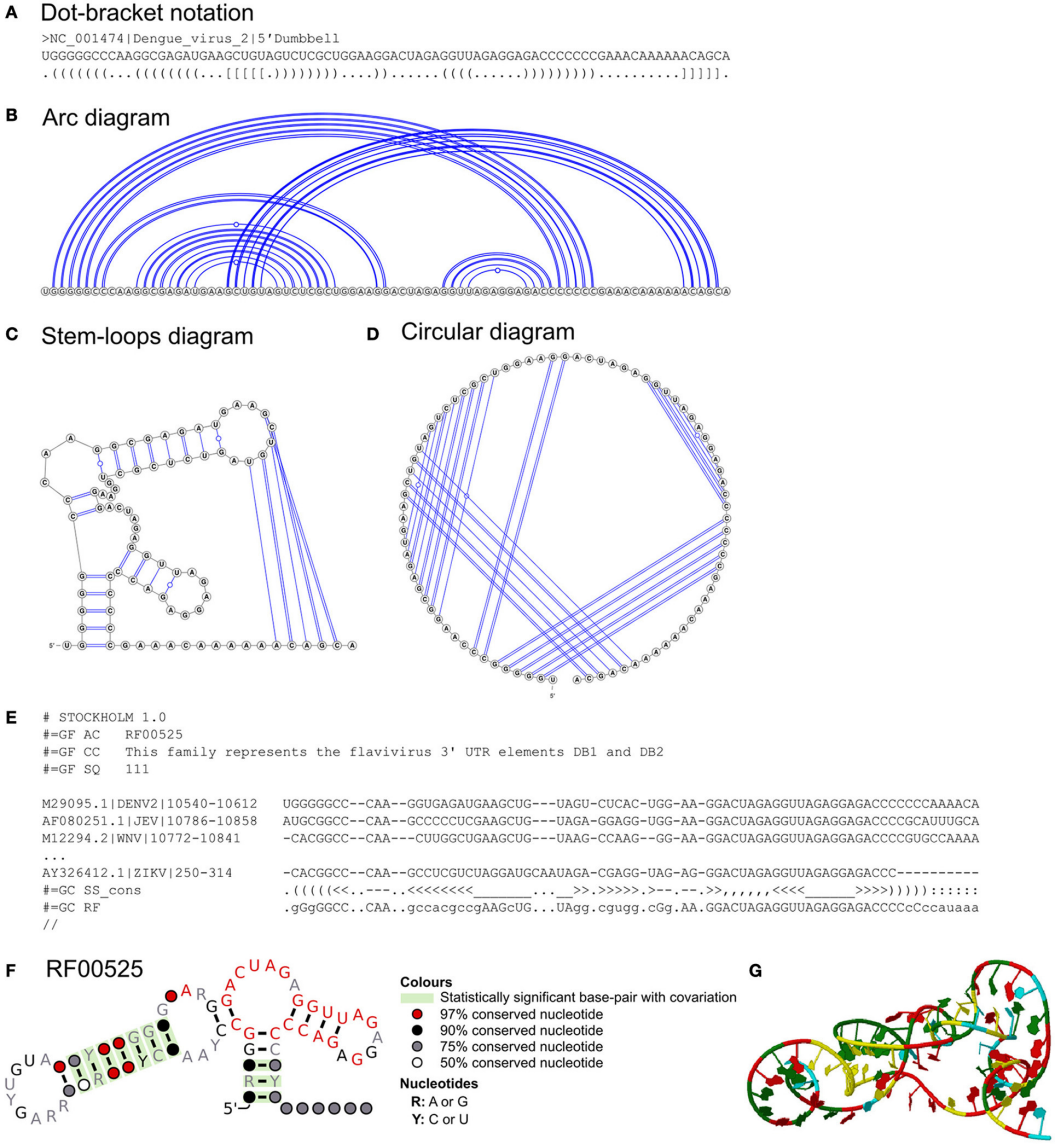
Dumbbell RNA structures of flaviviruses. Representations of 5′ dumbbell of dengue virus 2 in **(A)** dot-bracket notation, **(B)** arc, **(C)** stem-loops, and **(D)** circular diagrams. The diagrams are illustrated by VARNA (with pseudoknotted interactions). **(E)** The excerpts of the Stockholm file of the dumbbell elements (both 5′ and 3′ dumbbells) from Rfam. A Stockholm file consists of descriptions of the RNA structure of interest, multiple sequence alignment and consensus secondary structure in dot-and-bracket format. **(F)** Rfam model of the dumbbell structure assessed and illustrated by R-scape and R2R, respectively. **(G)** Representations of 5′ dumbbell of dengue virus 2 in 3D structure (modeled by SimRNAWeb; Magnus et al., 2016).

The conventional representation of an RNA structure is the 2D stem-loop diagram (Figure 2). However, the stem-loop diagram is not suitable to represent higher order interactions such as pseudoknotted interactions (Figure 2C, Table 1). In contrast, these tertiary interactions represented by the dot-bracket notation (Hofacker et al., 1994), and circular (Nussinov et al., 1978) and arc (Wattenberg, 2002) diagrams are easier to interpret (e.g., Figures 2A,B,D, respectively). These diagrams can be generated using VARNA, which requires dot-bracket notation as the input (Darty et al., 2009). Arc diagrams can also be created using R-CHIE, which is available as R package and web service (Lai et al., 2012).

However, these diagrams and dot-bracket notation normally represent one sequence at a time. Often common RNA elements are found in related sequences e.g., viral genotypes. Stockholm format is commonly used to represent the consensus RNA secondary structure of aligned sequences (Figure 2E; https://en.wikipedia.org/wiki/Stockholm_format). Stockholm format also stores some metadata e.g., the description of the aligned RNA sequences. R2R uses Stockholm file as the input to generate a novel stem-loop diagram of the consensus RNA secondary structure annotated with sequence conservation and covarying base-pairs (Figure 2F). Stockholm format and R2R stem-loop diagrams are both used by the Rfam database (see section on “Sources of Known RNA Structures”). Stockholm format file editors are available (Griffiths-Jones, 2005; Waterhouse et al., 2009).

### Bioinformatic tools

Many RNA structure prediction tools were initially released as command line software (Zuker, 1989). Biologists and virologists who are interested in using these would first learn the command line interface, this was and is a barrier for many researchers. However, where possible significant efforts have been made by developers to make their tools more readily available, as webservers (Backofen et al., 2017; Fallmann et al., 2017) or integrated graphic user interfaces (e.g., RNAStructure, or the Simple Sequence Editor, SSE; Simmonds, 2012; Bellaousov et al., 2013; Wang et al., 2017). For example, mfold which is the most cited RNA software in virology papers, was first released as a command line software in the late 1980s and became available as web interfaces in early 2000s (Zuker, 2003).

In contrast to folding one sequence at a time [single-sequence methods e.g., mfold/UNAfold (Zuker, 2003), RNAfold (Gruber et al., 2008)], a new generation of software such as LoCARNA (Smith et al., 2010) and RNAz (Gruber et al., 2007) work on multiple sequences (comparative methods). This alleviates the need of predicting RNA structures from related virus sequences one at a time and comparing them manually. Different methods vary in whether they align or fold first or do both simultaneously (Gardner and Giegerich, 2004).

The current range of functional RNA structures and prediction tools may seem intimidating (http://en.wikipedia.org/wiki/List_of_RNA_structure_prediction_software). However, many RNA structure prediction tools and RNA-RNA interaction prediction tools have been compared for use in different applications (Gardner and Giegerich, 2004; Gardner et al., 2005; Puton et al., 2013; Umu and Gardner, 2016). In general, comparative methods are more accurate than the older single-sequence methods (Puton et al., 2013).

Many of these powerful applications have been underused by virologists. For example, Infernal (INFERence of RNA Alignment; Nawrocki and Eddy, 2013) and CMfinder (Yao et al., 2006) that are based on both sequence and RNA secondary structure conservation allow sensitive detection of homologous RNA structures. A list of software that has been cited in selected virology publications is available (http://bioanalysis.otago.ac.nz/Lim2017.htm). Notably, these are beginning to include newer webservers which predict RNA 2D and 3D structures with high confidence.

Current methods often provide a 2D and oversimplified view of a certain sequence forming a single RNA structure. This is incorrect particularly in viral RNAs, where structures need to be transiently formed and melted (Moss et al., 2012a; Zhu and Meyer, 2015). This one-to-one sequence and structure relationship is also not true in many RNA viruses because they may exist in a quasispecies state where sequence space is sampled by high levels of replication error (Holmes, 2010; Lauring and Andino, 2010; Marz et al., 2014). Conservation in RNA structures but not the primary sequences across rapidly evolving species being particularly striking, e.g., the HIV frameshift site is one of the most conserved parts of the genome (Mathew et al., 2015).

## Know your enemy

**Figure 3:**
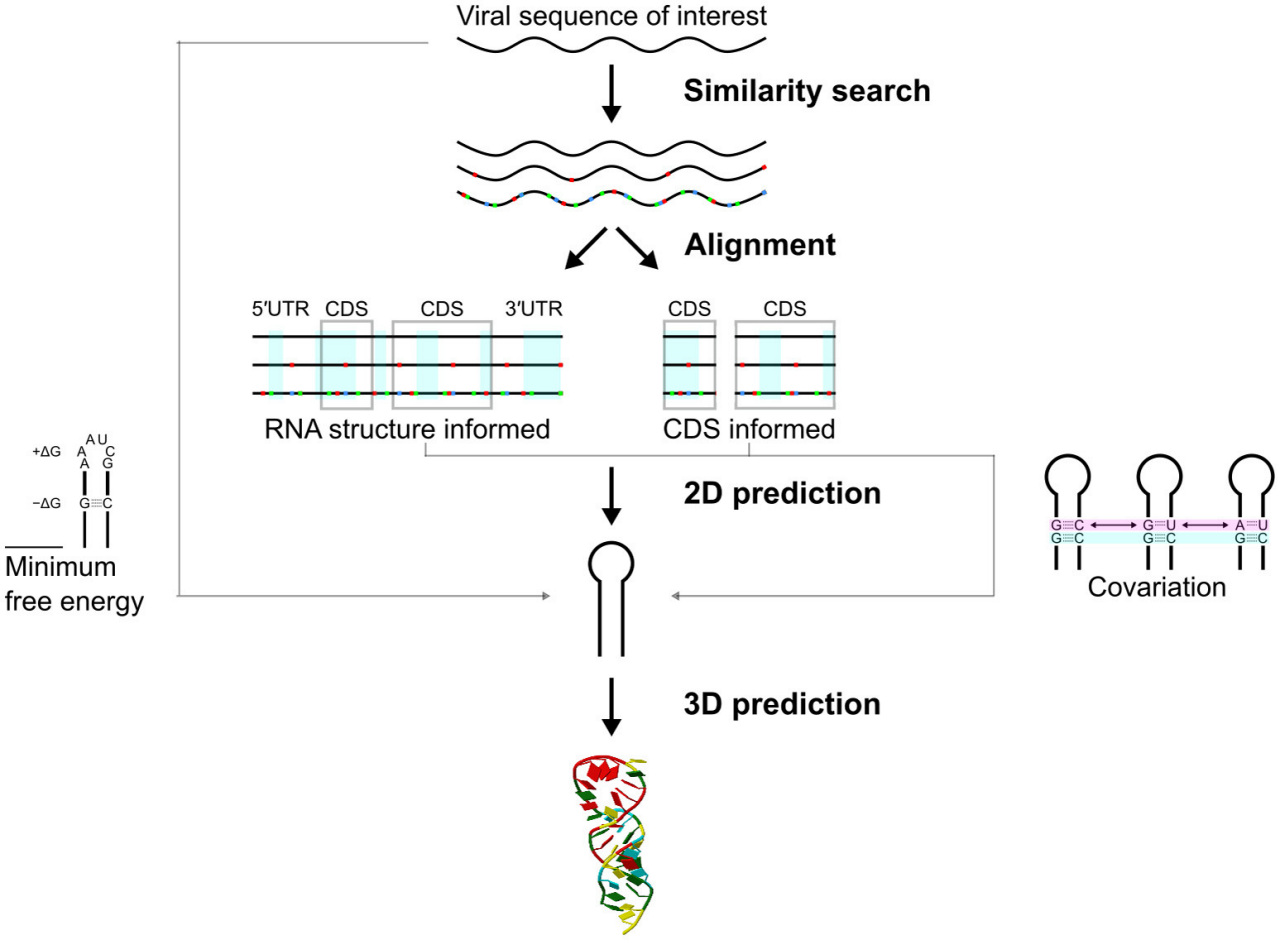
Approaches in prediction of structured RNA elements in RNA viruses. A virus sequence of interest can be matched to the NCBI/RefSeq database (see section “KNOW YOUR ENEMY”). A range of related sequences can be aligned using RNA structure informed and/or CDS informed approaches. Structured RNA elements of a virus are likely conserved in structure rather than primary sequence (red, blue, and green dots indicate mismatches). Secondary structures can be predicted from the aligned sequence. Covariation of a secondary structure can be tested statistically. Secondary structures can also be predicted directly using minimum free energy MFE) approach. RNA 3D prediction can also be done.

The starting point for RNA structure analysis is likely to be a complete (or partial) RNA genome (Figure 3). This could be a well-studied virus, or come from an outbreak of a new or emerging virus (e.g., SARS or Zika). The aim of these analyses is to further understand the biology of the viruses, and also to identify drug or vaccine targets.

Viral RNA elements have been identified as antiviral targets due to conservation of sequence and functions that are distinguishable from the host (Panjaworayan and Brown, 2011; Chen et al., 2014; Cardno et al., 2015; Le Grice, 2015; Hermann, 2016; Hilimire et al., 2017). For example, the internal ribosome entry sites (IRES) of hepatitis C virus (HCV) is targeted by benzimidazole (Dibrov et al., 2012). Another example is the HIV frameshift site, that has characteristics distinct from human frameshift sites (Cardno et al., 2015; Mathew et al., 2015; Hermann, 2018). In addition, double stranded viral RNA structures could be targeted by the host innate immune response, e.g., through Protein kinase RNA-activated (PKR) (Hartmann, 2017).

Targeting these viral specific features requires understanding of both the viral genomic sequence and its functional and sequence variation constraints—including gene structure and RNA *cis*-elements (Newburn and White, 2015; Hermann, 2018).

As a first step a similarity search on the NCBI/RefSeq database may not only allow identification of the virus, but also identify related viral sequences that could assist in predicting functional elements (Figure 3). Deep and accurate multiple sequence alignment is crucial in predicting likely RNA structures (Backofen et al., 2017; Fallmann et al., 2017). Specialized databases may also provide high quality sequence alignments to researchers, such as the LANL sequence databases for HIV, HCV, and hemorrhagic fever viruses (e.g., Ebola; Kuiken et al., 2012; Hatcher et al., 2017).

A novel virus can be classified according to the International Committee on Taxonomy of Viruses (ICTV) (King et al., 2011). The viral biology can be inferred if its species is well-characterized using published literature, and facilitated by general databases e.g., the ViralZone knowledgebase (Hulo et al., 2011) and specialized parts of the sequence databases e.g., NCBI Virus Variation Resource (Hatcher et al., 2017), RefSeq (O'leary et al., 2016), and NCBI Viral Genomes Resource (Brister et al., 2015). ICTV and ViralZone are further discussed in the next section “Virus Biology and RNA Structures.”

In conjunction with RNA structure analysis, potential coding sequences (CDS) can be predicted, in at least the three forward reading frames. This is an important step prior to prediction of RNA structures located in the coding sequence (Liu et al., 2009; Firth, 2014), for example frameshifting elements (Giedroc and Cornish, 2009). The beginnings and ends of these potential CDS are hotspots for RNA structures (Newburn and White, 2015).

If possible, alignments should be made to assist in identifying likely CDS and RNA structures (Firth, 2014; Figure 3). Similar sequences may be found with blastn, although non-coding similarity may be missed unless the initial hit size (word size) is reduced from the default of 11 to the greatest sensitivity available: 7. Alternatively, more sensitive local similarity search programs based on Smith-Waterman algorithm such as FASTA (SSEARCH; Lipman and Pearson, 1985; Pearson and Lipman, 1988) and SWIPE (Rognes, 2011) may be used, but are slower than blastn. FASTA is available through EMBL-EBI tools (https://www.ebi.ac.uk/Tools; Mcwilliam et al., 2013). Creating alignments for detection of elements within CDS can be facilitated by searching with the encoded protein (e.g., tblastn and tblastx). This will give greater sensitivity than blastn searches.

If it is known that the RNA regions encode for proteins (CDS) and/or contain RNA structures, alignment algorithms that consider this should be used [e.g., webPRANK (Löytynoja and Goldman, 2010) or R-Coffee (Taly et al., 2011), respectively; Figure 3]. RNA structures can also be detected in unaligned sequences, although these methods are more computationally intensive. Ideally, RNA primary sequence alignments should have dissimilarity of about 5–20% (Theis et al., 2015). Near identical aligned sequences may lack complexity that allows accurate RNA structure prediction and are not usually included in the prediction phase (see the success story on “RNA Structures in Coding Regions of Influenza A Virus”). However, the phenotype of a viable virus with a mutation in the structure may be informative (Kobayashi et al., 2016).

### Virus biology and RNA structures

Most RNA structures play *cis*-regulatory roles in various stages of the virus life cycle. Therefore, the functions of RNA structures can partly be inferred from their locations (Newburn and White, 2015). The RNA structures located near the 5′ end are mostly involved in replication and initiation of translation, such as the dimer linkage structure (DLS) of retroviruses (Johnson and Telesnitsky, 2010) and IRES of *Picornaviridae, Flaviviridae* in particular HCV and *Discistroviridae*, respectively (see section on “Internal Ribosome Entry Sites (IRES)”; Lee et al., 2017). Overlapping CDS may indicate frameshifts which would then direct the search to specific primary features, and nearby stem-loops or pseudoknots (Miras et al., 2017; see sections on “KNOW YOUR ENEMY” and “pseudoknots”). Whereas, RNA structures located near the 3′ end are often important in nuclear export of viral RNAs, such as the Rev response element (RRE) of human immunodeficiency virus (HIV) (Groom et al., 2009) and in replication, processing, or RNA stability (Newburn and White, 2015). However, other elements e.g., *cis*-acting replication elements (CRE) can be found in various genomic locations. For example, it is located at the 3′ end of HCV but the CDS of poliovirus (Tuplin et al., 2002; Dutkiewicz et al., 2016). Structured RNA elements in different locations of many viral genomes were reviewed in detail by Romero-López and Berzal-Herranz (2013); Brinton and Basu (2015); Newburn and White (2015); Nicholson and White (2015); Sagan et al. (2015); Madhugiri et al. (2016) and Fernández-Sanlés et al. (2017). For specific example of the functions and locations of RNA structures, see section on “RNA Structures in Barley Yellow Dwarf Virus (BYDV).”

Some guide to what structures to look for can also be obtained from the classification and biology of the virus of interest. ViralZone provides up-to-date information about viral biology, but it is protein and virus centered, rather than RNA structure focused (Hulo et al., 2011). As of June 2017, it documents the biology of 110 viral families, based on literature review, each entry is linked to Uniprot viral proteins. In ViralZone, summaries have been made under the section “Viral molecular biology: Transcription, replication, translation” (http://viralzone.expasy.org/915). This allows us to infer the viral molecular biology, which in turns provides some clues of what structural RNA elements to search for.

**Figure 4:**
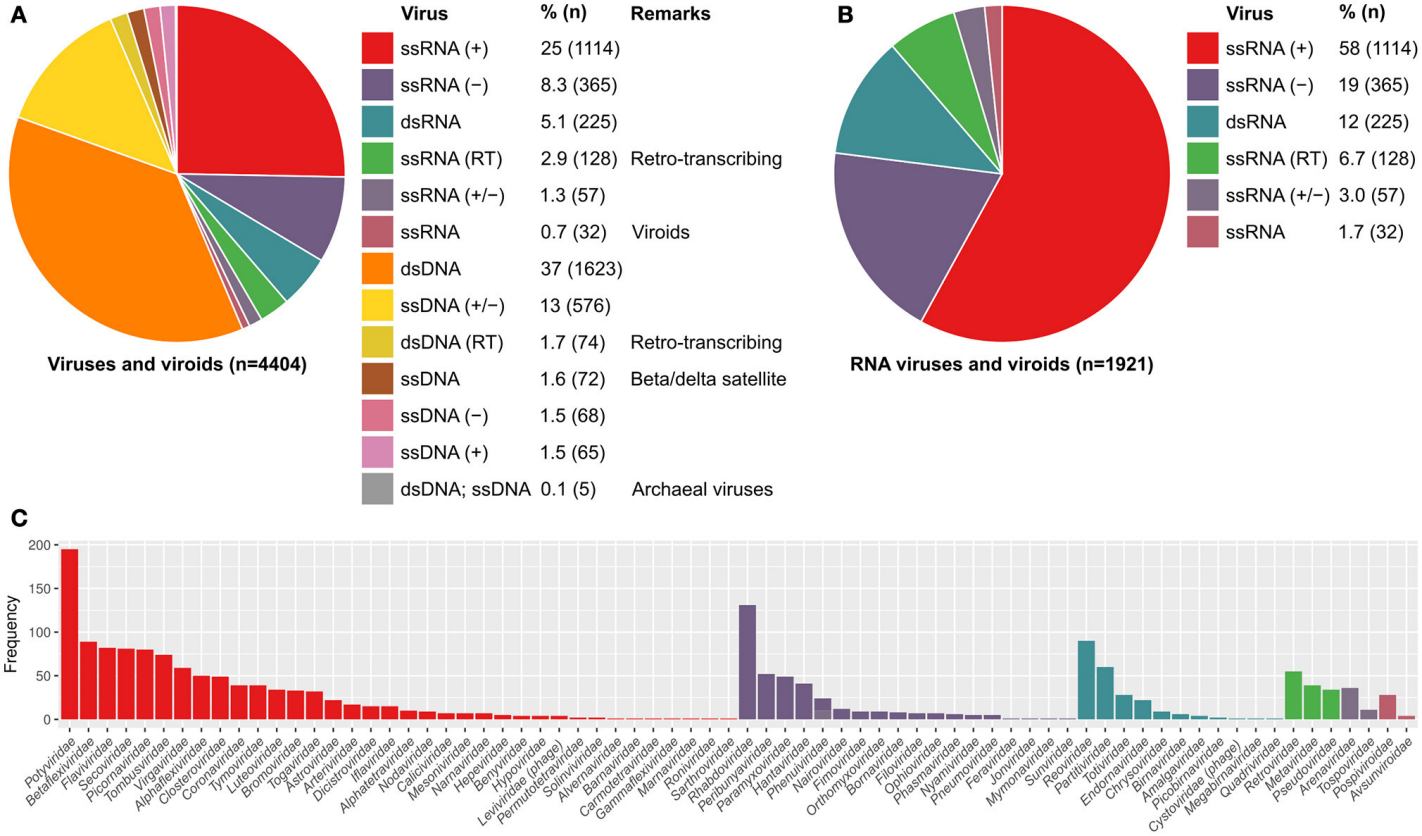
Proportion of known viruses and viroids based on the Baltimore classification, used in the ICTV database. **(A)** The genetic material of about 44% of known viruses and viroids is RNA. **(B)** About 58% RNA viruses and viroids are positive strand RNA viruses [ssRNA (+)] of which **(C)** *Potyviridae* are the largest family. RNA viruses are usually enriched with RNA structures. This is partly due to both the replication and transcription of eukaryotic RNA viruses occur in the cytoplasm, which are distinct from the host system and are driven by viral RNA elements. RNA virus transcripts therefore lack 5′-m^7^G-cap and are translated via unusual mechanisms such as internal ribosome entry site (IRES)-mediated translation and cap-independent translation. Only two RNA virus families are bacteriophages, namely *Leviviridae* and *Cystoviridae*, which are positive-sense single-stranded RNA and double-stranded RNA viruses, respectively.

Currently, the ICTV master species list (2016 v1.3) has the taxonomic classification of 4404 viruses and viroids, 44% of these are RNA viruses (Figures 4A,B). There are a total of 73 RNA virus families. Notably, over half (58%) of the RNA viruses are positive-sense single-stranded RNA viruses. RNA viruses are often enriched with RNA structures. This is partly due to the replication and transcription of RNA viruses occurring in the cytoplasm, which are regulated by viral RNA elements. The genomes and transcripts of some RNA viruses lack the 5-m^7^G (cap) requiring cap-independent translation (Simon and Miller, 2013). Indeed, some RNA viruses (e.g., picornaviruses) shutoff the host mRNA translation and use cap-independent translation such as IRES-mediated translation (Chase and Semler, 2012).

In contrast, over 99% of bacterial and archaeal viruses (bacteriophages) are DNA viruses (Figure 4A; ICTV master species list 2016 v1.3), although these may use RNA structures in their life cycles, notably as regulatory switches (Walsh and Mohr, 2011; Yang et al., 2014) and may have structured ncRNA (Hill et al., 2016). Only two RNA virus families infect bacteria, namely *Leviviridae* and *Cystoviridae*, which are positive-sense single-stranded RNA and double-stranded RNA viruses, respectively (Figure 4C). Several RNA bacteriophages are well-characterized such as MS2, Q, F1, and phi6. In particular, the 19-nucleotide MS2 packaging signal stem-loop of *E. coli* MS2 phage has been extensively studied. This high affinity MS2 packaging signal stem-loop is located at the ribosomal binding site of the replicase mRNA. Translation is inhibited upon the strong and specific binding of MS2 capsid protein (Peabody, 1990; Lim and Peabody, 1994; Stockley et al., 1995; Johansson et al., 1998). Recent studies indicate that other RNA viral genomes may have multiple structured capsid protein binding sites (Patel et al., 2017). The properties of MS2 have been exploited for various novel applications such as pull-down, tethering proteins to RNAs, RNA affinity purification, and live cell imaging of RNAs and protein-RNA interactions (Bardwell and Wickens, 1990; Bertrand et al., 1998; Coller et al., 1998; Graveley and Maniatis, 1998; Rackham and Brown, 2004).

### Sources of known RNA structures

**Figure 5:**
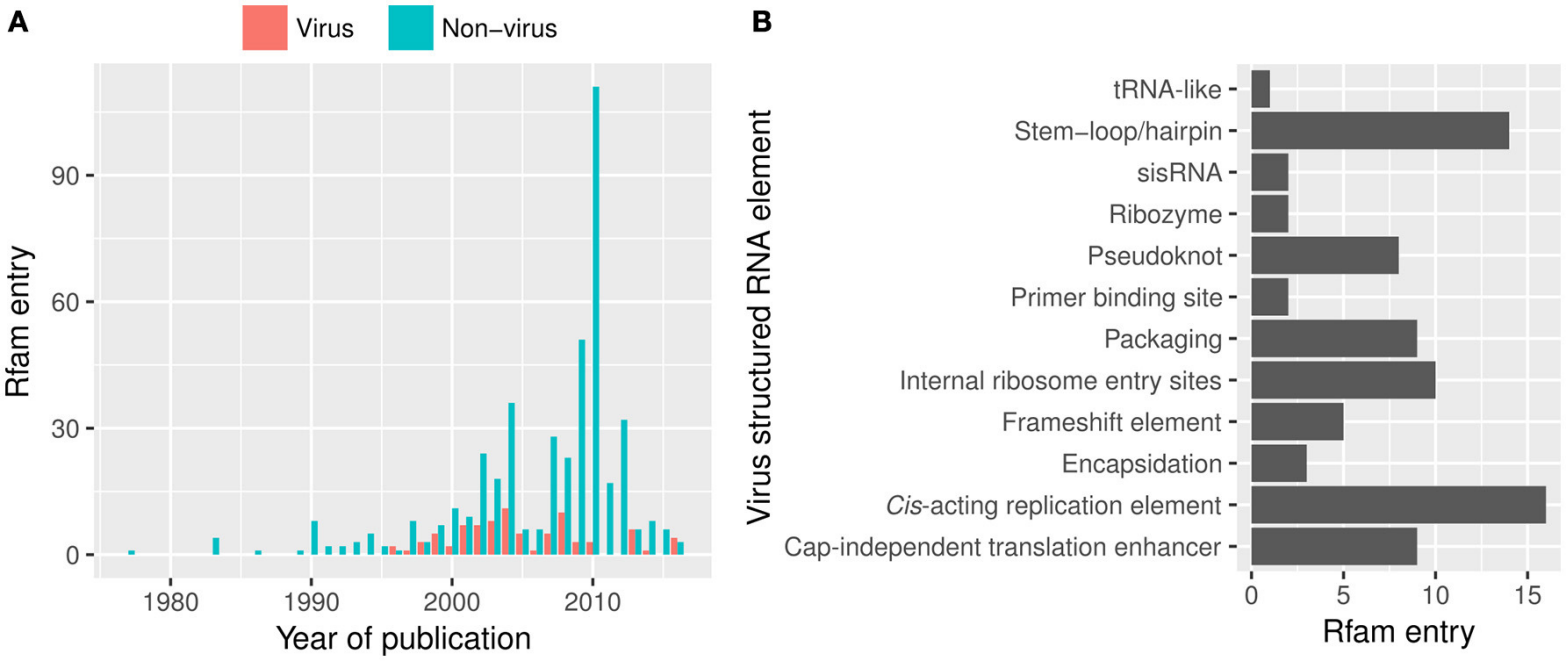
Viral structured RNA elements from Rfam 12.2. **(A)** The number of the structured RNA families published in journal articles over the years and **(B)** viral RNA families available in Rfam. However, the viral entries are likely overrepresented by RNA structures at the untranslated regions as those located in the coding sequence are often overlooked. sisRNA, stable intronic sequence RNA.

An example of useful resource that is not frequently cited by virus research articles is Rfam, the database of RNA structure families (Nawrocki et al., 2014). It contains over 105 viral RNA structural elements from both DNA and RNA viruses (Rfam 12.2, release January 2017; Figure 5). The most common viral RNA elements in Rfam are those in 3′UTRs (e.g., CITEs), 5′UTR (e.g., IRES), and packaging elements [e.g., packaging elements (*n* = 8) and *cis*-replication elements (*n* = 17, CRE) or encapsidation elements].

Importantly, the Rfam database can be used to annotate a viral sequence by searching for known RNA families with simple online tools (Nawrocki et al., 2014). Alternatively, the roles of novel RNA structures can be inferred by comparing them to the existing RNA families (Eggenhofer et al., 2013). Once characterized researchers can submit new RNA families to Rfam. Automated resources and guides for building families are available (Eggenhofer et al., 2016). Building these models may be facilitated by using combinations of software (Chen X. S. and Brown, 2012) in particular the WAR webserver (Torarinsson and Lindgreen, 2008) then be published as family descriptions online and/or in RNA Biology (Gultyaev and Olsthoorn, 2010; Chen A. and Brown, 2012; Lim and Brown, 2016).

Experimentally determined three dimensional RNA structures and descriptors of common structural elements (e.g., kink-turns, Table 1) are found in the Nucleic Acid Database (NDB) and related databases (Coimbatore Narayanan et al., 2014). These elements can be automatically included in homology based 3D predictions (Antczak et al., 2016).

In addition to this general database, there are specialized databases containing particular structural or functional classes of elements, some of which are overrepresented in viruses. The database of pseudoknots (Pseudobase++) contains 252 virus records (accessed in June 2017). IRESite contains 44 viral IRES entries (June 2017; Mokrejs et al., 2010). Recode contains many viral recoding sites, in particular RNA elements stimulating frameshifting and readthrough (Bekaert et al., 2010).

## Viral success stories

To illustrate the key concepts of RNA structure prediction, in this section we review the approaches used to successfully study the RNA structures located in flaviviruses, influenza, and BYDV. Common types of RNA structures are illustrated in Figure 1 and described in Table 1.

In choosing these examples we note that different concepts and approaches should be used in predicting the RNA structures located in the CDS in contrast to UTRs (Figure 3). RNA structures in the CDS have often been overlooked, and have only been discovered recently in some well-characterized viruses (see below). We will therefore review a successful story begun by several careful bioinformatics analyses of the CDS of the influenza A virus (Moss et al., 2011). As in experimental approaches, these examples show that independent approaches and tools have been required to accurately predict an RNA structure.

### RNA structures in the 3′UTRs of flaviviruses

Flaviviruses are positive-sense single-stranded RNA viruses, e.g., the mosquito-borne Dengue and Zika viruses. The RNA structures of flaviviruses have recently been reviewed (Villordo et al., 2016; Fernández-Sanlés et al., 2017). The sequences and RNA structures of the 3′UTRs of flaviviruses have been studied over three decades. Earlier studies found that the 3′UTR sequences of flaviviruses are highly divergent immediately after the stop codon, but remarkably similar at the distal region of the 3′UTR (Mandl et al., 1993; Wallner et al., 1995; Poidinger et al., 1996). Earlier computational and biochemical studies also found that a long stable hairpin structure at the 3′UTRs of flaviviruses (3′-LSH) had a similar structure, but not sequence (Grange et al., 1985; Brinton et al., 1986; Hahn et al., 1987; Mandl et al., 1993; Wallner et al., 1995; Shi et al., 1996). Remarkably, the dumbbell RNA structures of the 3′UTRs of flaviviruses were first discovered by Proutski et al. and Rauscher et al. in 1997 using only computational approaches (Proutski et al., 1997; Rauscher et al., 1997) whereas Rauscher et al. used the Vienna RNA package and a comparative approach including multiple sequence alignment (Gruber et al., 2008; Lorenz et al., 2011). Rauscher et al. found many covarying base-pairs in these structures, providing compelling evidence for RNA structure conservation. For example, there are 10 statistically significant covarying base-pairs in flavivirus dumbbell structures (RF00525; Figures 2E,F) currently annotated in Rfam (Nawrocki et al., 2014). Covarying base-pairs of RNA structures and the depth of aligned sequences can be statistically tested using R-scape (Rivas et al., 2017).

The RNA structures of flavivirus 3′UTRs were subsequently refined and proposed as H-type pseudoknots (tertiary structures) by Olsthoorn and Bol (2001) using mfold with suboptimal folding (e.g., 5′ dumbbell of dengue virus 2 flavivirus; Figures 2A–D). The structures of these flavivirus RNA elements were recently validated by SHAPE (Selective 2′-Hydroxyl Acylation analyzed by Primer Extension) chemical probing, mutation analysis and X-ray crystallography (Manzano et al., 2011; Chapman et al., 2014a,b; Villordo et al., 2015; Akiyama et al., 2016).

More importantly, many independent experiments have successfully uncovered their complex roles which have clinical implications. For example, deletion of the dengue virus 5′ dumbbell structure attenuates the virus, generating vaccine candidates that have been used for clinical testing (Whitehead et al., 2007). It is shown that assembly of the host RNA helicase DDX6 and other proteins at the dumbbell structures of dengue virus 2 is required for virus replication (Manzano et al., 2011; Ward et al., 2011). These 3′UTR structures also protect flaviviral subgenomic RNAs (sfRNAs) from the host Xrn1 5′-3′ exonuclease digestion (Pijlman et al., 2008). These sfRNAs are pathogenic and important in regulating viral life cycle (Manzano et al., 2011; Chapman et al., 2014b; Akiyama et al., 2016) and have been targeted by specific antiviral oligomers (Zhang et al., 2008).

### RNA structures in coding regions of influenza A virus

Influenza A virus is a zoonotic virus that infects a wide range of mammals and birds (Shi et al., 2014). It is a negative-sense single-stranded RNA virus that has an eight-segment genome. Moss et al. (2011) undertook a careful analysis of complete genomes of Influenza A strain H5N1 and H1N1 infecting human, avian and swine from NCBI Virus Variation Resource (Hatcher et al., 2017). This enabled them to discover many putative structured RNA elements located in the CDS of Influenza A virus (Moss et al., 2011).

To create multiple sequence alignment, Moss et al. first translated the CDS into protein sequences. The aligned protein sequences were then converted back to nucleotide sequences. They scanned the aligned CDS for putative RNA structures using RNAz (Gruber et al., 2007). They used sliding windows of 120-nucleotide, with 10-nucleotide steps. This allows rapid prediction of local RNA structures in the 120-nucleotide windows of the whole aligned sequence. They also detected synonymous (for the encoded protein) sites in the aligned CDS using SSE (Simmonds, 2012), that were constrained during evolution. These codon-based alignments detect synonymous constraints, possibly due to the presence of structured RNA elements. This is based on the assumption that synonymous substitutions in a CDS are restricted by base-pairing required for RNA folding, but such constraints could also be due to primary sequence conservation in RNA (or DNA).

Alternatively, codon-based alignment could have been be done using webPRANK (Löytynoja and Goldman, 2010) or Codon Alignment (HCV sequence database; Kuiken et al., 2005). Significant synonymous constraint sites of aligned CDS can also be detected using FRESco (Sealfon et al., 2015) or synplot2 (Firth, 2014). Many automated alignments of viral genomes are available using codon based alignments in searches for conserved RNA structures or overlapping CDS (Hofacker et al., 2004; Firth and Brown, 2006; Firth, 2014).

Moss et al. (2011) predicted and refined the potential structured regions using RNAalifold (Bernhart et al., 2008), and Dynalign (Mathews and Turner, 2002). Pseudoknots were predicted using DotKnot (Sperschneider and Datta, 2010). Notably, a predicted pseudoknot located in the virus segment 2 genome was subsequently shown to be consistent with that of chemical probing results (Priore et al., 2015). The predicted RNA structures near the splice junctions of M1/M2 and NS1/NEP transcripts were also validated experimentally and/or found to be important for the virus viability (Moss et al., 2012b; Jiang et al., 2016).

To improve the power of detecting putative RNA structural elements, subsequent studies focused on specific genes/genome segments, namely HA (surface glycoprotein hemagglutinin; Gultyaev et al., 2016), M (Kobayashi et al., 2016), and NP (nucleoprotein; Gultyaev et al., 2014; Soszynska-Jozwiak et al., 2015) using deep multiple sequence alignment. Indeed, new structured RNA elements have been continuously discovered. For example, Kobayashi et al. (2016) analyzed 1884 sequences of M gene from 88 Influenza A virus subtypes. Similar to the Moss et al. (2011) approaches, they scanned the deeply aligned CDS for potential RNA structured regions and synonymous variations using SSE (Simmonds, 2012). Prediction of the RNA structured regions was based on UNAfold MFE algorithm implemented in SSE (Simmonds, 2012). They predicted RNA structures on the regions with both low MFE and synonymous substitution rate using RNAalifold. Remarkably, disrupting the base-pairs of the RNA structures located at the 5′ and 3′ ends of M gene using synonymous mutations reduced the infectivity and attenuated the virus, respectively (Kobayashi et al., 2016).

In sum, these studies highlight the strength of comparative approach in detection of RNA structures in the CDS. Different comparative methods used by these studies can be compared and combined to achieve better results. However, these powerful comparative approaches are underutilized by virologists.

### RNA structures in barley yellow dwarf virus (BYDV)

**Figure 6:**
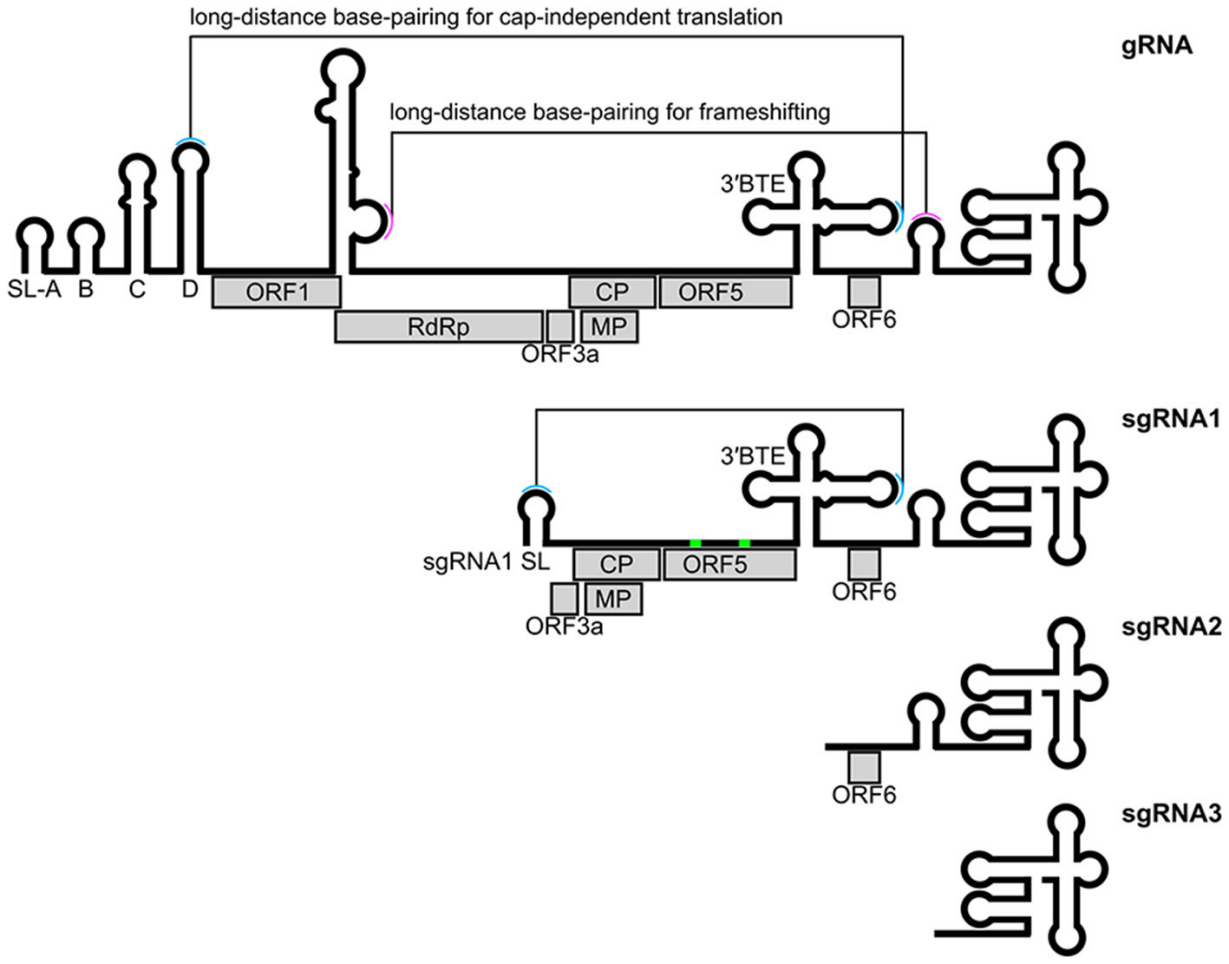
Structured RNA elements of BYDV. CP readthrough elements are shown in green. BYDV, barley yellow dwarf virus; CP, coat protein; BTE, BYDV-like translation element; gRNA, genomic RNA; MP, movement protein; ORF, open reading frame; RdRp, RNA-dependent RNA polymerase; sgRNA, subgenomic RNA; SL, stem-loop.

Luteoviruses including BYDV are important plant pathogens. BYDV infects barley, maize, oats, rice, and wheat, causing yellowing and dwarfing of the hosts (D'arcy and Domier, 2000). It is arguably the viral genome with the greatest range and diversity of RNA structures. The type member BYDV-PAV has a 5.7 kb positive stranded RNA genome, and three coding and non-coding subgenomic RNAs (Figure 6). From the initial sequence of the genome and prediction of ORFs, it was likely that it would have require multiple non-canonical translation events to make key proteins—sgRNA expression, frameshift, readthrough, leaky-scanning, and cap-independent translation (Miller et al., 1988). Much careful experimental work indicated that these events require both structured and loosely-structured RNA elements (Miras et al., 2017). Studies on frameshifting and cap-independent translation in BYDV showed that both local and distant sequences are required for full activity (Miller et al., 2015). Some of these are used as illustrations in the following sections.

## Viral RNA structures

### Stem-loops/hairpin

Most computational analysis will begin by predicting stem-loops but these can be built up into more complex structures (Figure 1, Table 1). Prediction accuracy is increased if an alignment of sequences that fold into the same structure is used (Gorodkin et al., 2014). When a stem-loop is predicted, attention should also be given to bulges, internal, and terminal loops (Figure 1). For example: terminal loops may form more stable structures e.g., tetraloops; and be sites of RNA or protein interaction; apparent bulges may form non-canonical pairs (e.g., A-G); and unpaired bases are more likely to form sites of interactions (Lozano et al., 2016). Modeling of loops is more difficult, but can be done thermodynamically (Sloma and Mathews, 2016), by using similarity to known elements e.g., tetraloops, or known experimentally determined folds (Theis et al., 2015; Roll et al., 2016; Phan et al., 2017).

### Pseudoknots

**Figure 7:**
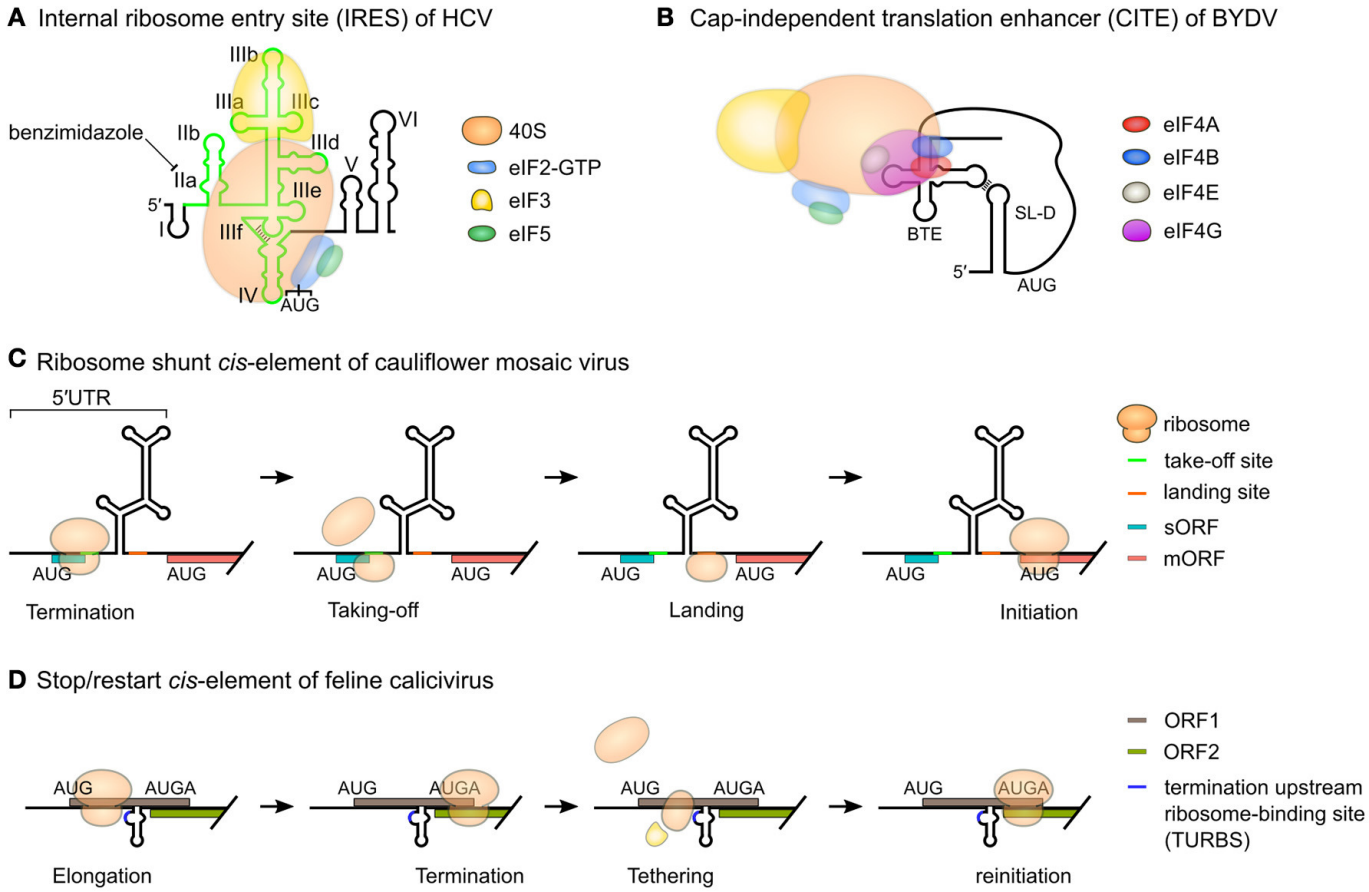
Functions of viral RNA structures. Viral structured RNA elements are important in viral replication, transcription and translation. Many RNA viruses hijack the host translation machinery and utilize unusual translation mechanisms for protein synthesis. **(A)** Internal ribosomal entry site (IRES) of HCV recruits eIF3 and 43S preinitiation complex to promote a cap-independent translation mechanism called IRES-mediated translation. The domains II to IV of HCV (light green) are the key RNA motifs of IRES. This unusual translation mechanism can be inhibited by benzimidazole by targeting the domain IIa. The domain IIIf is a pseudoknot. **(B)** Cap-independent translation enhancer (CITE) of BYDV (BYDV-like translation enhancer, BTE) recruits eukaryotic initiation factors and 40S ribosomal subunit, forming long-range interactions with stem-loop-D (SL-D) to promote translation. **(C)** Unusual translation mechanisms can also occur in some polycistronic viral RNAs. The 5′UTR of cauliflower mosaic virus is long and highly structured. The highly structured region contains multiple upstream AUGs. A highly structured 5′UTR with multiple upstream AUGs could inhibit translation of the main open reading frame (mORF) of a eukaryotic mRNA. Cauliflower mosaic virus overcome this problem with ribosome shunt *cis*-element. A ribosome first translate the small ORF (sORF) at the viral 5′UTR. During translation termination, the ribosome dissociates but the take-off site (the sequence surrounding the termination codon) induce ribosome shunting. This allows the ribosome to bypass the highly structured region of the 5′UTR, land on the landing site, followed by translation of the mORF. **(D)** Feline calicivirus contains two ORFs with a slightly overlapping sequence AUGA. A structured motif called stop/restart *cis*-element located upstream of AUGA permits effective reinitation and translation of the second ORF. A termination upstream ribosome-binding site (TURBS) located in the RNA structure allows tethering of 40S ribosomal subunit and eIF3. This promotes reinitiation of the second ORF.

In some cases the terminal loop may form additional “pseudoknot” base-pairs (Table 1, Figure 1C, and Figures 2A–D). These are most easily visualized on arc and circular diagrams of the suboptimal RNA secondary structures (Figures 2B,D). Pseudoknots are found in specific parts of the viral genome involved in translation and replication (Brierley et al., 2007; Atkins et al., 2016), such as the domain IIIf of the HCV IRES (Figure 7A) and the dumbbell structures of dengue virus 2 (Figure 5), respectively.

The most common virus frameshift, is −1 frameshift, which often has a stimulatory pseudoknot(s). This frameshifting was first discovered in a retrovirus, Rous sarcoma virus (*gag/pol* frameshift; Jacks and Varmus, 1985). Many viral frameshifts have now been characterized [reviewed in detail by Atkins et al. (2016)]. Frameshifting elements consist of a slippery site, a spacer (~5–9 nucleotides) and a frameshift stimulator (stem-loop or pseudoknot) [reviewed in detail by Giedroc and Cornish (2009) and Firth and Brierley (2012)]. However, frameshift stimulators are highly diverse (Chung et al., 2010). In some cases, non-canonical base-pairs e.g., base triples (Chen et al., 2017) and long-range base-pairs may be required for −1 frameshifting (e.g., in BYDV, Figure 6; Miras et al., 2017).

In the prediction of −1 frameshifting RNA elements, a slippery site is usually searched for between the two frames (zero and −1). This has a consensus sequence of “X XXY YYZ,” the zero frame codons are separated by spaces, X is an identical nucleotide, Y is either A or U, and Z is not G (Brierley et al., 2007). A ribosome first encounters the slowly decoded codons of the slippery site. The stalled ribosome then “slips” one nucleotide backward (−1 frame; XXX YYY Z) to resume translation elongation. In most cases, a frameshift stimulator downstream (e.g., RNA structure) of the slippery site is required for physiological frameshifting. Although such structure is dispensable in at least one virus, Hibiscus latent Singapore virus (Niu et al., 2014).

Pseudoknots are often predicted by visual inspection from studying the local secondary structures following these slippery sites. Software has also been used, for example, the H-type pseudoknot structure modulating −1 frameshifting in Japanese Encephalitis virus was successfully predicted using PknotsRG (now part of pKiss; Janssen and Giegerich, 2015) and experimentally validated (Melian et al., 2009). This was confirmed in an independent study on a vaccine strain that harbors a synonymous mutation that abolishes the RNA structure (Sun et al., 2012). However, *de novo* pseudoknot computational prediction remains challenging, and current tools are <5% accurate (Leamy et al., 2016). This may be improved by including experimental data (Hajdin et al., 2013).

These ribosomal frameshifting sites can be predicted specifically using KnotInFrame (Theis et al., 2008). Others have used more general software, e.g., RNA Shapes Studio (Janssen and Giegerich, 2015) as was done for Zika virus, or combination of prediction programs as was used to predict a functional pseudoknot in West Nile virus (Moomau et al., 2016). Models in 3D can be built of pseudoknots e.g., using MC-Sym as was recently done for Venezuelan equine encephalitis virus (Kendra et al., 2017).

### Kissing hairpins

Kissing hairpins (also known as kissing-loops or kissing stem-loops) are formed from the base-pairing between the loop of two stem-loops (Table 1, Figure 1). Many kissing hairpins are related to virus replication or transcription (You and Rice, 2008; Ganser and Al-Hashimi, 2016).

The first viral kissing hairpins were discovered in enteroviruses (plus strand viruses), namely poliovirus and coxsackievirus B3 (Pilipenko et al., 1992). These structures are located at the 3′UTR of an enterovirus genomic RNA and required for synthesis of the viral negative strand RNA template (Dutkiewicz et al., 2016). These kissing hairpins are formed by base-pairing of two adjacent stem-loops which are known as X and Y motifs. Interestingly, the primary sequence of these motifs are conserved only in certain enterovirus subgroups but the Y motif variants were shown to be interchangeable between poliovirus and coxsackievirus B3 (Zoll et al., 2009).

Another well-studied example is the retroviral dimerization initiation sites (DIS). This structure is involved in dimerization of virus genomic RNAs, which is a critical step in retroviral replication (Paillart et al., 2004). The “kissing” begins at the DIS of two virus genomic RNAs prior to encapsidation (Mailler et al., 2016).

DotKnot (Sperschneider and Datta, 2010), pKnots (Rivas and Eddy, 1999), pKiss (Theis et al., 2010), and pAliKiss (Janssen and Giegerich, 2015) could also be used to predict such kissing hairpins. However, these are limited to predicting intramolecular kissing interactions.

### Cloverleaf/tRNA-like structures

A tRNA-like structure harbors a four-way junction—three stem-loops (a cloverleaf), and in viral structures may also contain additional pseudoknots (Figure 1, Table 1). In enteroviruses, a cloverleaf structure known as *ori*L is involved in viral replication (Prostova et al., 2017). It is located at the 5′ leader of the plus strand genomic RNAs (Dutkiewicz et al., 2016). The cellular PCBP [poly(rC)-binding protein] and viral protein 3CD^pro^ binds to two different stem-loops of *ori*L, forming a replication complex. Other proteins could also bind to *ori*L (Prostova et al., 2017). A cloverleaf structure is also formed in the negative strand RNA template, the kissing interaction of the hairpin loops, within the cloverleaf structure is required for viral genomic RNA synthesis (Melchers et al., 1997).

Many positive strand plant viruses have such tRNA-like structures in the 3′UTRs of genomic RNAs (Dreher, 2010). Most viral tRNA-like structures are aminoacylated (e.g., by Val, His, or Tyr), mimicking cellular tRNAs to regulate translation. A recent study proposed that these tRNA-like structures can also act as mobile elements in plant by promoting transport of viral transcripts via phloem sap (Zhang et al., 2016). These tRNA-like structures are amenable to both modeling and experimental 3D determination. For example, the tRNA-like structure of Tobacco mosaic virus was recently solved by X-ray crystallography (Colussi et al., 2014), this tRNA-like structure has multiple additional upstream pseudoknots. Indeed, viral tRNA-like structures discovered to date have variable sequence, length, and structures (Dreher, 2010).

### Long-range intra-molecular interactions

The elements considered above form mainly local structures. These local structures may form in nascent RNA (Meyer, 2017) or be stabilized by protein or RNA binding. Local structures can be predicted using appropriate windows of sequence (e.g., 80–200 bases) and it is also practical to analyse local alignments of similar lengths (Lange et al., 2012).

Long-range interactions from over a few hundred bases to >26 kb do occur in RNA viruses, but are difficult to predict accurately. Challenges include: there are many possible interactions; likely complex structures (e.g., pseudoknots); structures will form co-transcriptionally limiting interactions; and small molecules, proteins, RNAs and complexes (e.g., ribosomes) will bind and affect folding (Lai and Meyer, 2016; Napthine et al., 2016; Sun et al., 2017).

Methods based on MFE when applied to long RNAs (e.g., mfold on a viral genome) will tend to predict large structures with a large number of long-distance interactions—which should be viewed with caution. Indeed, experimentally determined structures of full length genomes show more local than long-range interactions e.g., HIV RNA has many local structures (Watts et al., 2009) but only five long-distance interactions (Fricke and Marz, 2016).

Specific tools have been developed to predict long-range interactions, e.g., LRIscan, with 14 of 16 known long-distance interactions confirmed and plausible candidates from other viruses predicted (Fricke and Marz, 2016).

Efficient frameshifting, in addition to the local frameshifting elements (e.g., pseudoknots), may require long-range interactions (Nicholson and White, 2014). These have been well-characterized in BYDV (Paul et al., 2001; Barry and Miller, 2002) and red clover necrotic mosaic virus (RCNMV; Tajima et al., 2011) and involve long-range kissing interactions (Figure 6).

### Inter-molecular interactions

Some viral RNAs also form structures with other RNAs, both viral and cellular. Notable examples are viral RNA dimerization elements (see subsection “Kissing Hairpins”), co-packaging elements, or interactions during translation with the rRNA in the ribosome (Deforges et al., 2015; Angulo et al., 2016). Co-packaging of multiple segments of RNA may suggest that inter-molecular interactions occur, for example in RCNMV the loop of origin of assembly stem-loop on RNA2 interacts with on RNA1 (Newburn and White, 2015). Specific software e.g., RNAhybrid can be used to predict such inter-molecular interactions (Rehmsmeier et al., 2004).

## Functions of viral RNA structures

The roles of many structured RNA elements of viruses have been studied in detail. Some examples are discussed in this section.

### Internal ribosome entry sites (IRES)

Viral RNAs are not always capped, this means that they have evolved specific mechanisms to enhance cap-independent translation. The RNAs of many viruses contain large structured IRES, to promote this. Well-characterized examples are found in picornaviruses and HCV (Lee et al., 2017; Figure 7A). The IRES recruits ribosomes near or directly to the translation initiation codons of viral mRNAs, bypassing the need for the cap-binding complex. This allows the virus to manipulate the host translation machinery by inhibition or proteolytic cleavage of host eukaryotic initiation factors (eIFs). Translation of viral mRNAs is possible even during the host translation shutoff (Lee et al., 2017).

However, viral IRES are one of the most challenging structural elements to predict and characterize. This is because IRES are complex and diverse, often consisting of multiple stem-loops and/or pseudoknots (Dreher, 2010; Lozano et al., 2016). Limited progress has been made in development of automated pipelines for IRES prediction, however, two specialized webservers are available, namely VIPS (Hong et al., 2013) and IRESPred (Kolekar et al., 2016). Both VIPS and IRESPred predict IRES based on known IRES sequences and structures. In particular, IRESPred looks for the binding sequence motifs of small subunit ribosomal proteins. Alternatively, a combination of tools, in particular BLAST, Pfold, Centroid Fold, mfold, and pKiss have also proven to be useful in IRES prediction (Asnani et al., 2015, 2016).

### Ribosome shunt *cis*-elements

Ribosome shunting consists of a series of unusual translation events (Figure 7C). A ribosome first initiates at a small ORF (sORF) and terminates right before a large RNA structure. The large ribosomal subunit dissociate but the small subunit bypasses the RNA structure, docks on a landing site and resumes scanning. The ribosome can then reinitiate even at a non-AUG codon. The shunt elements were discovered in DNA viruses, first in the pararetrovirus cauliflower mosaic virus (Fütterer et al., 1990, 1993), then a retrovirus, prototype foamy virus (Schepetilnikov et al., 2009), and a plant RNA virus, rice tungro spherical virus (Pooggin et al., 2012).

To predict the *cis*-elements driving ribosome shunting, several key characteristics of these elements have been taken into account. These elements are located in a long, highly structured 5′UTR of the virus genomic RNA that has multiple upstream AUGs. These 5′UTR features appear to inhibit translation of the main ORF(s). The sORF(s) precede the large RNA structure is involved, whereas the following upstream AUGs are folded up in a large RNA structure. This RNA structure has a stable base-pairing at the stem base. The shunt take-off site (sequence around the sORF termination codon) and landing site are expected to be conserved between closely related viruses or co-evolved viruses. For example, the shunt *cis*-elements are remarkably similar between a pair of co-evolved viruses, a RNA picorna-like virus, rice tungro spherical virus and a DNA pararetrovirus, rice tungro bacilliform virus (Pooggin et al., 2012).

### Cap-independent translation enhancers (CITEs)

Cap-independent translation may be stimulated by local RNA structures but surprisingly in some cases also by long-distance base-pairing. This long-distance base-pairing has been well-characterized in several plant viral RNAs (Miras et al., 2017). For example, BYDV has a CITE located at the 3′ end (Figures 6, 7B). This element interacts with a stem-loop located at the 5′UTR (long-range kissing interactions) to promote cap-independent translation (Miller et al., 2015). Other viral genera also use long-distance base-pairing or interaction with rRNA (Deforges et al., 2015). Published models of two such complex structures have been made using RNA2D3D (Mccormack et al., 2008) and MC-Sym (Wang et al., 2011; Newburn and White, 2015).

### Stop/restart *cis*-elements

Some viruses use unusual mechanisms to reinitiate after translation of a long CDS. These stop/restart or termination-reinitiation mechanisms were initially found in Caliciviruses (Figure 7D) and then Influenza B viruses (reviewed in detail by Powell, 2010). These mechanisms allow effective translation of both the first and second ORFs of a viral mRNA, producing two distinct functional proteins (Zinoviev et al., 2015). These mechanisms require several *cis*-regulatory elements that can be partially structured, and these may interact with other RNAs e.g., the 18S rRNA.

These mechanisms are distinct from the mechanisms utilizing upstream ORFs (uORFs), or programmed ribosomal frameshifting (Miras et al., 2017). In eukaryotic mRNAs, including viral ones, uORFs are commonly found to repress translation of the mORFs (Hellens et al., 2016; Zong et al., 2017). These regulatory uORFs are usually short and therefore producing only small peptides (Hellens et al., 2016; Starck et al., 2016). Whereas, in ribosomal frameshifting, only one protein is produced with the use of two overlapping ORFs (Atkins et al., 2016).

The stop-start *cis*-elements in Caliciviruses and Influenza B viruses are found between −84 and the start codon of the second ORF (Powell, 2010; Zinoviev et al., 2015). These *cis*-elements consist of a termination upstream ribosome-binding site (TURBS) and a stop/restart site. TURBS consists of a motif 1 (18S rRNA complementary site), and motif 2 and 2^*^ (likely base-pairing and structured). Motif 1 is loosely structural to allow tethering of small ribosomal subunit for reinitiation whereas motif 2 and 2^*^ could form an RNA structure that enhances translation of the second ORF (Lee et al., 2017).

Recently, stop/restart *cis*-elements were found in helminthosporium victoriae virus 190S. These elements consist of a H-type pseudoknot and an A**UGA** stop/restart site (start and stop codons are underlined and bolded, respectively; Li et al., 2015). This pseudoknot was successfully predicted using HPknotter (Huang et al., 2005). Disruption of the tertiary base-pairs abolishes translation of the second ORF (Li et al., 2015).

To predict stop/restart *cis*-elements, one could first look for slightly overlapping ORFs with a stop/restart site. However, these *cis*-elements also enable translation of a synthetic, non-overlapping second ORF effectively within a range of 40 nucleotides downstream of the first ORF (Ahmadian et al., 2000; Napthine et al., 2009; Zinoviev et al., 2015). Motif 1 (18S rRNA complementary site) is likely present between −84 and the start codon of the second ORF. A RNA structure may also found within the region. However, suboptimal RNA structures could also be present (Napthine et al., 2009).

## Challenges and limitations

This review has presented examples where virology research has been enhanced by the appropriate use of bioinformatic methods for RNA structure prediction. These examples highlight how computer predictions were used in conjunction with experimental tools for functional studies. Some of computational tools and resources are generally applicable to RNA structure prediction whereas others are specific to virology. Additional prediction tools are continually becoming available (Backofen et al., 2017; Miao and Westhof, 2017; Miao et al., 2017).

However, some challenges remain for the application of newer RNA structure tools in virology (Table 1). Some of these are being addressed by user friendly suites and tools becoming available as noted throughout this review and listed in the companion website (http://bioanalysis.otago.ac.nz/Lim2017.htm). In addition, specialized workshops and training may facilitate the use of these RNA tools e.g., The EMBO Practical Course on Computational RNA Biology course material available online (https://bibiserv.cebitec.uni-bielefeld.de/EMBO-RNACourse/).

## Author contributions

Both authors have made a substantial, direct and intellectual contribution to the work, and approved it for publication.

### Conflict of interest statement

The authors declare that the research was conducted in the absence of any commercial or financial relationships that could be construed as a potential conflict of interest.
